# In Vitro Impact of Fluconazole on Oral Microbial Communities, Bacterial Growth, and Biofilm Formation

**DOI:** 10.3390/antibiotics12091433

**Published:** 2023-09-11

**Authors:** Louise Morais Dornelas-Figueira, Antônio Pedro Ricomini Filho, Roger Junges, Heidi Aarø Åmdal, Altair Antoninha Del Bel Cury, Fernanda Cristina Petersen

**Affiliations:** 1 Department of Prosthodontics and Periodontology, Piracicaba Dental School, University of Campinas, Piracicaba 13414-903, SP, Brazil; l.dornelas@dental.ufl.edu (L.M.D.-F.); delbelcury@gmail.com (A.A.D.B.C.); 2 Institute of Oral Biology, Faculty of Dentistry, University of Oslo, 0372 Oslo, Norway; roger.junges@odont.uio.no (R.J.); h.a.amdal@odont.uio.no (H.A.Å.); 3 Department of Physiological Sciences, Piracicaba Dental School, University of Campinas, Piracicaba 13414-903, SP, Brazil; pedroricomini@gmail.com

**Keywords:** fluconazole, microbiota, *Streptococcus*, biofilm, antimicrobials

## Abstract

Antifungal agents are widely used to specifically eliminate infections by fungal pathogens. However, the specificity of antifungal agents has been challenged by a few studies demonstrating antibacterial inhibitory effects against *Mycobacteria* and *Streptomyces* species. Here, we evaluated for the first time the potential effect of fluconazole, the most clinically used antifungal agent, on a human oral microbiota biofilm model. The results showed that biofilm viability on blood and mitis salivarius agar media was increased over time in the presence of fluconazole at clinically relevant concentrations, despite a reduction in biomass. Targeted PCR revealed a higher abundance of *Veillonella atypica*, *Veillonella dispar,* and *Lactobacillus* spp. in the fluconazole-treated samples compared to the control, while *Fusobacterium nucleatum* was reduced and *Streptococcus* spp were not significantly affected. Further, we tested the potential impact of fluconazole using single-species models. Our results, using *Streptococcus mutans* and *Streptococcus mitis* luciferase reporters, showed that *S. mutans* planktonic growth was not significantly affected by fluconazole, whereas for *S. mitis*, planktonic growth, but not biofilm viability, was inhibited at the highest concentration. Fluconazole’s effects on *S. mitis* biofilm biomass were concentration and time dependent. Exposure for 48 h to the highest concentration of fluconazole was associated with *S. mitis* biofilms with the most increased biomass. Potential growth inhibitory effects were further tested using four non-streptococcal species. Among these, the planktonic growth of both *Escherichia coli* and *Granulicatella adiacens* was inhibited by fluconazole. The data indicate bacterial responses to fluconazole that extend to a broader range of bacterial species than previously anticipated from the literature, with the potential to disturb biofilm communities.

## 1. Introduction

The oral cavity is colonized by numerous microorganisms, including bacteria, fungi, archaea, protozoa, and viruses. Together, they form what is known as the oral microbiota. The coexistence of these microorganisms relies on their synergistic and antagonist interactions, resulting in a balanced microbial community that maintains potentially harmful microorganisms numbers at low levels [1]. However, stressors can disturb this microbial balance, leading to a condition called dysbiosis [2]. Dysbiosis results in an altered microbial community where potentially pathogenic microorganisms become more prevalent, increasing the risk of disease. Further, commensals play an important role in the establishment and balance of the microbiota; once this dynamic balance is disrupted, infections take place [3]. One of the most important stressors affecting microbial communities is the use of antimicrobials agents [1,4].

Antimicrobials play a crucial role in the treatment and control of infectious diseases. They include antifungal agents such as fluconazole, one of the most effective and prescribed agents for antifungal prophylaxis and for the treatment of oropharyngeal candidiasis in HIV-positive patients, neonatal, and *Candida* spp. infections [5,6,7,8,9,10]. Fluconazole has been used successfully to treat local diseases, such as vaginal candidiasis, oropharyngeal and esophageal candidiasis, and *Candida* spp. urinary tract infections; but also, to treat systemic infections including candidemia, disseminated candidiasis, coccidioidomycosis, and cryptococcal meningitis [6,11,12].

Fluconazole is a triazole that inhibits the cytochrome P450 enzyme lanosterol demethylase (14α-demethylase) involved in the ergosterol biosynthesis pathway in fungi, thus inhibiting cell membrane formation [5]. It is available mostly as enteral and intravenous preparations, but also as a mouthrinse or suspension for local infections [13,14]. The clinical efficacy of systemic fluconazole in preventing and treating oropharyngeal and esophageal candidiasis is attributed to the relatively high concentrations achieved in salivary secretions following oral administration [15].

The impact of antimicrobials in the human microbiota is an area that is receiving increasing attention due to the association of microbial community imbalances with different diseases and the alarming levels of antimicrobial resistance. Antifungals have the potential to disturb the balance of microbial communities in the gut [16] and promote diarrhea and nausea. In animal models, the disturbance of fungal populations leading to gut microbiota dysbiosis has been associated with the induction of host pro-inflammatory responses [17]. Also, increasing studies indicate that certain antifungal drugs, in particular imidazoles, may have direct antimicrobial activity against selected bacterial pathogens and commensals of the gut [16]. For antifungal triazoles, including fluconazole, antimicrobial activity against gut microbes has not been observed using stringent cutoffs [16]. It is possible, however, that fluconazole may have weak inhibitory activity, as observed for *Streptomyces lividans,* a soil bacterium also found in the gut [18]. Such studies indicate that antifungals may potentially promote dysbiosis in complex microbial communities by a third and unexplored mechanism, namely through antibacterial effects. 

In contrast to the gut, the negative effects of antimicrobials on the microbiota of the upper digestive tract are less understood. This is a field of increased interest due to the association of oral microbiome dysbiosis with various autoimmune, inflammatory, and neoplastic conditions [19,20,21]. Also, the microbial community of the upper digestive tract represents a reservoir of antibiotic resistance genes and bacterial pathogens [22,23,24]. In this study, we aimed to investigate the possible effect of fluconazole on a complex human microbial community using an oral microbiota biofilm model, and on the growth of selected species of streptococcal and non-streptococcal colonizers of the oral cavity. 

## 2. Materials and Methods

### 2.1. Experimental Design

For the oral microbiota study, non-stimulated saliva was collected from 6 healthy subjects aged between 25 and 35 years old. Subjects were asked to abstain from drinking and eating 2 h prior to saliva collection. The study was conducted in accordance with the Declaration of Helsinki and approved by the Norwegian Regional Ethics Committee (REK20152491) for studies involving human samples. The saliva was first collected, centrifuged, pooled, and added to the bottom of the wells of 24-well plates to form a salivary pellicle, as previously described [25]. After that, the saliva-coated plates were sterilized in UV light (2500 µJ/cm^2^ for 30 min), followed by the addition of SHI media containing pooled salivary microbiota (2 µL/mL) to the wells and incubation in an anaerobic chamber at 37 °C for 24 h. The detached cells and old media were then carefully removed, and fresh SHI medium containing fluconazole (Cayman Chemical Company; Ann Arbor, MI, USA. 11594) was added to the wells. Fluconazole stock solutions prepared by suspending 5 mg fluconazole in 5 mL 100% ethanol were stored at −20 °C. Two concentrations of fluconazole were tested in the microbiota model: 2.56 μg/mL, corresponding to common concentrations in saliva after oral administration (SA) [15]; and 2000 μg/mL, which is the concentration used in mouthrinses (MRs) [13]. Samples without fluconazole, but with the same concentration of the dissolving agent, were included as negative controls. Three independent experiments with two to three replicates were performed. After 24h incubation, the biofilm supernatants were analyzed for potential effects of fluconazole on metabolic activities by measuring final pH. Biofilms were resuspended in PBS and used for (1) measurement of potential effects on microbial viability by determining colony forming units (CFUs) on agar plates after 48 h incubation at 37 °C in 5% CO_2_ atmosphere, (2) measurement of bacterial and fungal DNA by quantitative PCR, (3) measurement of changes in microbial composition by targeted PCR, and (4) biofilm dry biomass by weight measurements. 

To evaluate the effects of fluconazole on *Streptococcus* spp. planktonic growth, we used the type of strain of *S. mitis* and the *S. mutans* strain UA159, each engineered to harbor an ldh-luciferase reporter system, as described below. The impact of fluconazole on *S. mitis* biofilm growth was also investigated. In this assay, biofilms were formed in 96-well flat bottom plates with fluconazole at different concentrations: saliva concentration 2.56 μg/mL (SA), peak plasma concentration 4.39 μg/mL (1×), twofold peak plasma concentration 8.78 μg/mL (2×) and at mouthrinse concentration 2 mg/mL (MO). The microorganisms were incubated at 37 °C and the relative light units (RLU) and optical density (OD) were measured every 30 min. Negative control groups without fluconazole were included in all experiments. Three independent experiments with two to three replicates were performed. To elucidate the effect of fluconazole on *S. mitis* biofilm formation, biofilms were grown in tryptic soy broth (TSB) supplemented with 0.2% glucose in 5% CO_2_ at 37 °C. After 6, 24, and 48 h, the biofilms were harvested and CFUs, biomass (dry weight), and pH were measured. 

### 2.2. pH Assay

The pH in the supernatant of the oral microbiota samples was measured at 6, 24, and 48 h growth with the aid of a pH meter (pH 3110 Einzelgerät—WTW^®^) calibrated with pH standards of 4.0 and 7.0.

### 2.3. Viability Assay

To estimate the viability of microorganisms in the oral microbiome samples, the counts of the colony forming unit (CFU) were performed. Aliquots of 100 μL of the biofilm suspension were tenfold serially diluted in PBS until 10^−7^. Two separate drops of 20 μL of the dilution were plated on both blood agar and mitis salivarius agar (MSA) and incubated at 37 °C, 5% CO_2_ for 24 h. The count was carried out with the aid of a stereoscopic microscope.

### 2.4. Biomass

Dry weight was used to evaluate the potential impact of fluconazole on oral microbiome biomass. An aliquot of the suspension was transferred to previously weighed microtubes. Later, an amount of 3 times absolute ethanol was added to the tubes and stored at −20 °C for 20 min. Then, the tubes were centrifuged at 10,000× *g* for 5 min at 4 °C, the supernatants were discarded, and the biofilms were centrifuged under heat and vacuum to dehydrate the samples. Dry weight was determined by the difference in the final and initial microtube weight. 

### 2.5. Real-Time PCR

To evaluate the concentration of bacterial and fungal DNA in the microbiota, total DNA was extracted using the Zymo Quick-DNA Fungal/Bacterial Micro prep extraction kit (Zymo Research) and quantified by real-time PCR using Zymo DNA quantification kits for bacteria/fungi (Femto Bacterial and fungal DNA Quantification kit, Zymo Research) following the manufacturer’s protocol. The samples used for DNA extraction were from 200 µL of the 1mL re-suspended biofilms. For the PCR reactions, we used 3 µL of undiluted DNA for measuring the amount of fungal DNA and 3 µL of 1000-fold dilutions for measurement of bacterial DNA, added to 18 µL of the Femto™ Fungal/Bacterial qPCR Premix. “No template” controls were also included. The AriaMx Real-Time PCR software system was used for calculations (Agilent Techonologies, Santa Clara, CA, USA, 95051).

Real-time PCR was further used to investigate the impact of fluconazole on microbial composition, using primers specific to *Streptococcus* spp., *Lactobacillus* spp., *Veillonella atypica*, *Veillonella dispar*, *Prevotella intermedia,* and *Fusobacterium nucleatum* (Table 1). The samples used were the same as above, but with the DNA adjusted to have similar concentrations in the reactions. The final PCR reaction volume was 25 μL, comprising 12.5 μL Maxima SYBR Green/ROX qPCR Master Mix (2×) containing Maxima Hot Start Taq DNA Polymerase, dNTPs, and SYBR Green I in an optimized PCR buffer with ROX passive reference dye, 3 ng DNA template, 0.4 μM forward and reverse primers. The thermal cycling program was as follows: 95 °C for 10 min; and then 40 cycles consisting of denaturation at 95 °C for 15 s, primer annealing at 55 °C for 30 s, and primer extension at 72 °C for 30 s. The thermal cycle was finalized with a cycle of 95 °C for 1 min, 55 °C for 30 s, and 95 °C for 30 s. Dissociation curves were prepared immediately after the last PCR cycle. Relative fold changes were calculated using the 2^−∆∆Ct^ method [26].

### 2.6. Construction of Luciferase Reporters

Lactate dehydrogenase is essential in the ATP-generating pathway of streptococci. Its promoter activity provides both viability and metabolic status information in response to antimicrobials [27]. The luciferase reporter in *S. mutans* (SM120; Table 1) was constructed using the pair of primers FP514 and FP515 to amplify the lactate dehydrogenase promoter region (p*ldh*) of *S. mutans* UA159 (Table 1). The primers were designed with the *Nhe*I and *Bam*HI restriction sites on the 5′ end. The resulting amplicon was digested with the referred restriction enzymes and ligated to pFW5-luc [28], a luciferase reporter plasmid carrying a spectinomycin resistance cassette that works in both Gram+ and Gram- bacteria. Cloning in *E. coli* was performed as described previously [29], and a plasmid with the correct insert was then purified and used to transform *S. mutans* UA159 via natural transformation [30]. Insertion in the *S. mutans* chromosome was confirmed by selection of positive mutant colonies performed in tryptic soy agar plates with spectinomycin (500 μg/mL) and verified phenotypically using the luciferase growth assay. Design and construction of mutant MI048 in *S. mitis* has been previously described [31].

### 2.7. Luciferase Reporter Assay

The luciferase reporter assay was as described before [31]. Briefly, overnight cultures of *S. mitis* MI048 and *S. mutans* SM120 reporter strains were adjusted to the final concentration of approximately 1 × 10^8^ CFU·mL^−1^ (OD_600_ 0.1) [31]. The microorganisms were cultured in 96-well flat bottom plates (Nunc Thermo Scientific) with tryptic soy broth (TSB) supplemented with 0.2% glucose and different fluconazole concentrations. A negative control group without fluconazole was also included, as well as blanks containing pure medium. In addition, 10 µL room temperature 1.0 mM luciferin solution (Synchem, Felsberg-Altenberg, Germany) was added to each 200 µL culture. The cells were incubated at 37 °C and the relative light unit (RLU) and optical density (OD) were measured at various time intervals during growth in a microplate reader (Synergy HT; BioTek, Winooski, VT, USA).

### 2.8. Planktonic Growth

To better elucidate whether fluconazole’s effect on bacterial growth was restricted to *Streptococcus* spp., a planktonic growth assay was conducted with *Escherichia coli* (DH10BTM), *Granulicatella adiacens* (clinical isolate), *Lactobacillus crispatus* (ATCC 33820) and *Lactobacillus salivarius* (ATCC 11741). Overnight cultures were diluted to OD600 0.1 and were incubated at 37 °C until they reached stationary phase. Optical density (OD) was measured at various time intervals in a Bioscreen C system reader (Lab systems, Helsinki, Finland).

**Table 1:** Bacterial strains and primers used in this study.

Strain or Primers	Description or Sequence ^a^	Source or Reference ^b^
**Strains**		
CCUG31611	*Streptococcus mitis* type strain	CCUG
UA159	*Streptococcus mutans* wild-type reference strain	[32]
MI048	CCUG31611, but p*_ldh_*-luc; Spc^r^	[31]
SM120	UA159, but but p*_ldh_*-luc; Spc^r^	This study
**Primer pairs**		
FP514 ^c^ forward	agctagcACCGCATCAATTGTTTCTCC	pFW5-*luc*
FP515 ^d^ reverse	aggatccCACCATCACCAACAAGGATG	
FP1067 forward	CCATGAAGTCGGAATCGCTAG	16S rRNA
FP1068 reverse	GCTTGACGGGCGGTGT	
FP2387 forward	GATACATAGCCGACCTGAG	*Streptococcus* spp. New England Biolabs
FP2388 reverse	CCATTGCCGAAGATTCC	
FP2297 forward	GTACAGTTGCTTCAGGACGTATC	*Streptococcus* spp. [33]
FP2298 reverse	ACGTTCGATTTCATCACGTTG	
FP2341 forward	TCTCTTGTTGAAGAATTAGAACGC	*Veillonella atypica* [34]
FP2342 reverse	GTGTAACAAGGGAGTACGGACC	
FP2389 forward	CCGTGATGGGATGGAAACTGC	*Veillonella dispar* [34]
FP2390 reverse	CCTTCGCCACTGGTGTTCTTC	
FP2303 forward	CTCAAAACTAAACAAAGTTTC	*Lactobacillus* spp. [35]
FP2304 reverse	CTTGTACACACCGCCCGTCA	
FP2383 forward	CGGTCTGTTAAGCGTGTTGTG	*Prevotella intermedia* [36]
FP2384 reverse	CACCATGAATTCCGCATACG	
FP2381 forward	GGATTTATTGGGCGTAAAGC	*Fusobacterium nucleatum* [33]
FP2382 reverse	GGCATTCCTACAAATATCTACGAA	

^a^ Spc^r^, spectinomycin resistance. ^b^ CCUG, Culture Collection University of Gothenburg; ATCC, American Type Culture Collection. ^c^ *Nhe*I restriction site underlined. ^d^ *Bam*HI restriction site underlined.

### 2.9. S. mitis Biofilm Assay

We further analyzed the effects of fluconazole on biofilm formation by Streptococcus mitis. For that, we used the S. mitis strain used in the planktonic experiments described above. Frozen stock cultures stored at −80 °C were used for growth in blood agar plates. After 24 h, colonies were transferred to tryptic soy broth (TSB) supplemented with 0.2% glucose and incubated in 5% CO_2_ at 37 °C. The inoculum was adjusted to the final concentration of OD 0.1 and the biofilms were formed in 96-well flat bottom plates with fluconazole, using the same concentrations as for planktonic growth. The biofilms were assessed for bacterial viability (CFU counts) and biomass (dry weight) at 6, 24, and 48 h. 

### 2.10. Statistical Analyses

The results were analyzed using GraphPad software. The assumptions of equality of variances and normal distribution of errors were evaluated for each variable. When the data were not normally distributed, they were transformed. The statistical analysis was performed using one-way ANOVA, having fluconazole concentration as the study factor. Post-ANOVA comparisons were performed using the Tukey test.

## 3. Results

### 3.1. Oral Microbiota Biofilm 

**Figure 1:**
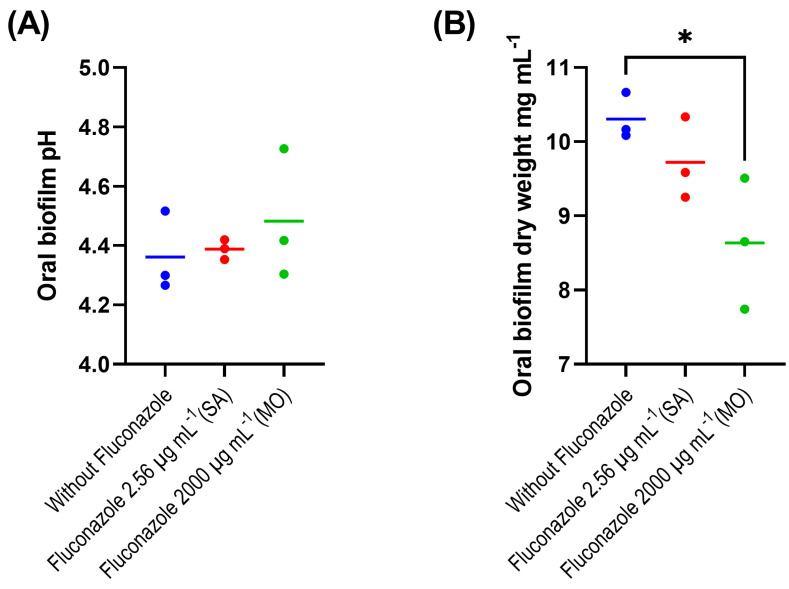

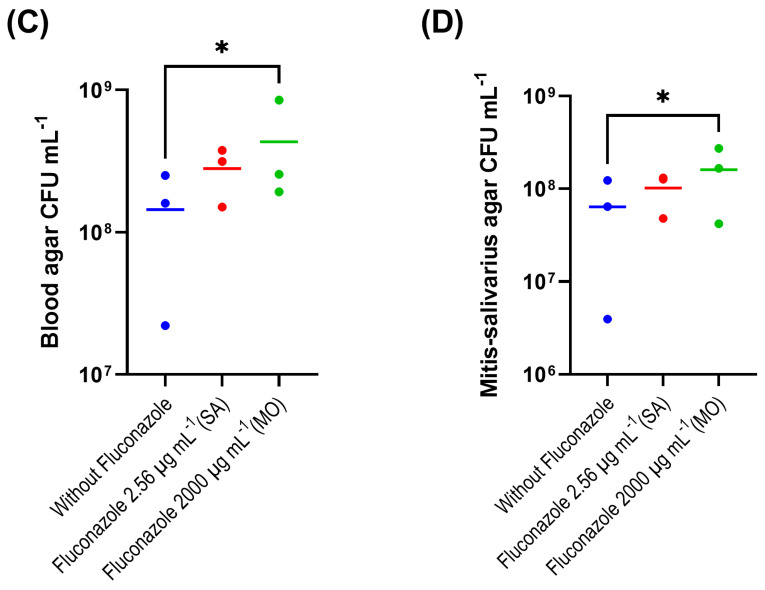
Oral microbiota biofilm assay showing pH (**A**), biomass (**B**), CFU counts on blood agar (**C**) and on mitis salivarius agar (**D**); 24 h biofilms were exposed to fluconazole at 2.56 μg·mL^−1^ (SA—salivary concentration) and 2000 μg·mL^−1^ (MO—mouthrinse concentration). Samples without fluconazole were included as control. The horizontal lines represent mean values from three independent experiments with three parallels each. Mean values from each of the parallel samples are represented by colored circles; * indicates statistical difference (*p* < 0.05) between groups; one-way ANOVA followed by the Tukey’s post hoc test.

The evaluation of the fluconazole effects on oral microbiota showed that the drug had no significant effect on the pH of biofilm supernatants collected at 24 h after treatment, despite a tendency for lower pH in the absence of fluconazole (*p* > 0.05) (Figure 1A). We also found that the highest concentration of fluconazole (2000 μg·mL^−1^) reduced biofilm biomass (*p* < 0.05) (Figure 1B). In addition, this concentration was associated with increased viable counts (CFUs) on both blood agar and mitis salivarius agar compared to the control, indicating an effect of fluconazole on the overall microbial community viability (blood agar plates), including *Streptococcus* spp. (mitis salivarius agar plates) (*p* < 0.05) (Figure 1C,D).

**Figure 2:**
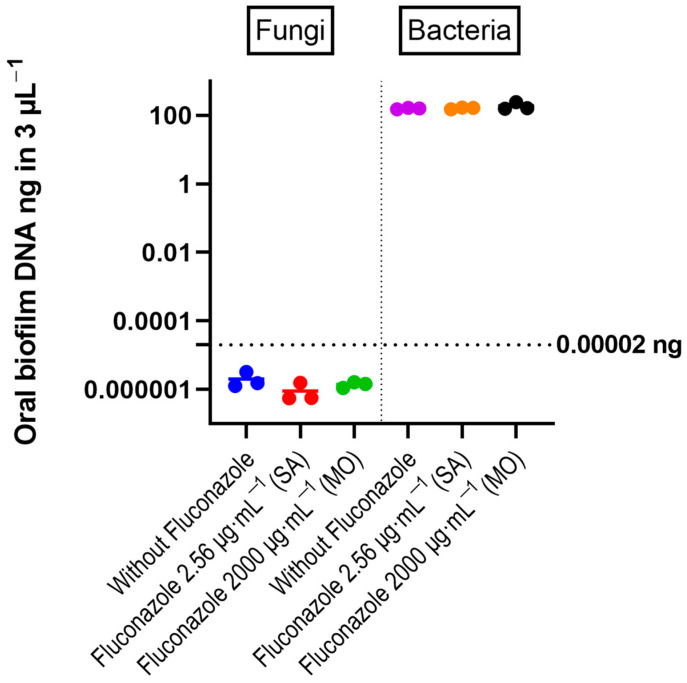
DNA amount of bacteria and fungi in the oral microbiota biofilms, determined by quantitative RT-PCR. The individual values from three experiments are shown as colored circles. Horizontal bars correspond to the mean values. The horizontal line at 0.00002 ng indicates the threshold recommended for reliable quantification in the RT-PCR reaction (Zymo Femto kit). One-way ANOVA test revealed no significant differences between control and fluconazole-treated samples.

**Figure 3:**
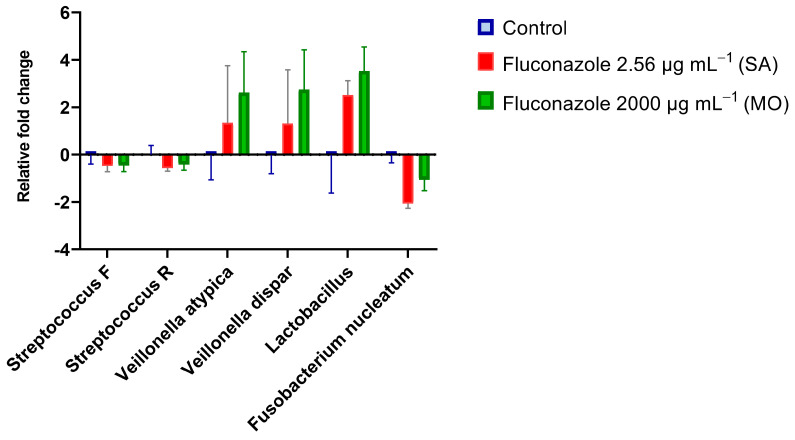
Real-time PCR results expressed by log2 relative fold changes (2^−∆∆Ct^) show the impact of fluconazole at 2.56 μg·mL^−1^ (SA), 2000 μg·mL^−1^ (MO), or without fluconazole exposure (negative control) on microbial composition.

qPCR results with quantitative sensitivity down to 20 femtograms did not reveal significant effects of fluconazole on total bacterial DNA (Figure 2). For fungal DNA, the amounts measured were below 20 fg, demonstrating that fungal DNA was more than nine orders of magnitude lower than the bacterial DNA in the samples. For the target PCR using three biological replicates for each group, only *Prevotella intermedia* was below the detection level. The largest changes were for *V. atypica*, *V. dispar*, and *Lactobacillus*, which were present in higher amounts in the fluconazole-treated samples compared to the control, while *F. nucleatum* was reduced in the treated group (Figure 3). 

### 3.2. Fluconazole Effect on Bacterial Planktonic Growth

**Figure 4:**
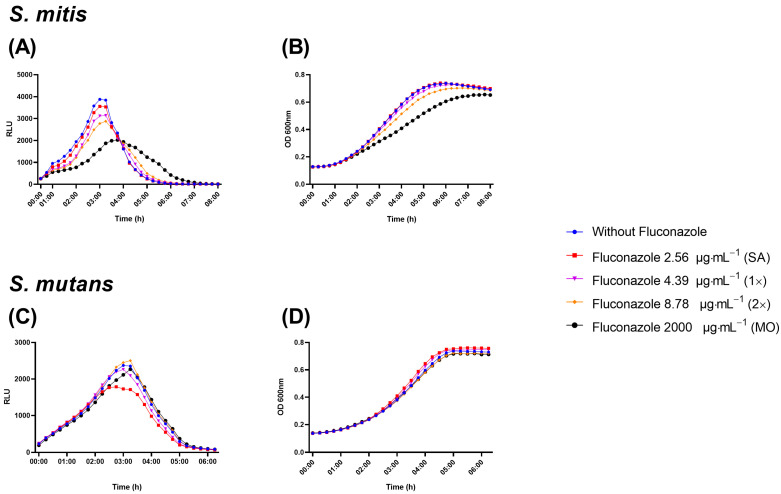
Real-time viability and metabolic activity assay showing *S. mitis* and *S. mutans* growth evaluated as relative light unit (RLU) by the expression of *_p_ldh-luc* reporter strains (**A**,**C**) and growth by optical density (OD; **B**,**D**). Bacterial cells were grown in the presence of fluconazole at 2.56 μg·mL^−1^ (SA), 4.39 μg·mL^−1^ (1×—peak plasma concentration), 8.78 μg·mL^−1^ (2×—twice peak plasma concentration), and 2000 μg·mL^−1^ (MO). Samples without exposure to fluconazole were included as negative control.

The luciferase assay uses a reporter for lactate dehydrogenase to reveal effects on metabolic activity in the presence of luciferin by different treatments [27] This assay revealed a dose-dependent reduction on luminescence by *S. mitis* in the presence of fluconazole (Figure 4A). In line with the inhibitory effect on metabolic activity, growth measured as optical density at 600 nm was also inhibited by fluconazole (Figure 4B). For *S. mutans*, no significant effects were observed by any of the two methods (Figure 4C,D). 

**Figure 5:**
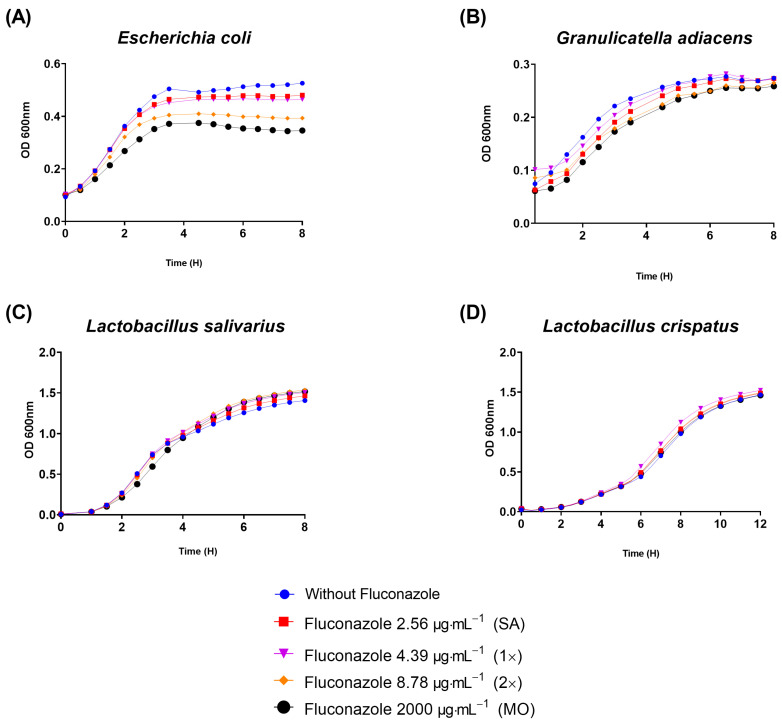
Planktonic growth results assessed by optical density (OD) showed that fluconazole at 2.56 μg·mL^−1^ (SA), 4.39 μg·mL^−1^ (1×), 8.78 μg·mL^−1^ (2×), and 2000 μg·mL^−1^ (MO) reduced planktonic growth of *E. coli* (**A**), retarded *G. adiacens* growth (**B**), and did not influence *Lactobacillus salivarius* and *Lactobacillus crispatus* viability (**C**,**D**).

To gain initial insights on whether the growth of other bacteria could be affected by fluconazole, four other species representing both Gram-negatives and Gram-positives were selected. Interestingly, fluconazole reduced the planktonic growth of the Gram-negative *E. coli* (Figure 5A) and delayed the growth of the Gram-positive *G. adiacens* (Figure 5B), while no effect was observed on the growth of *Lactobacillus salivarius* and *Lactobacillus crispatus*, both Gram-positives (Figure 5C,D). The results indicate that a spectrum of activity by fluconazole seems specific to certain bacteria, and that can include both Gram-positives and Gram-negatives.

### 3.3. Effect of Fluconazole on S. mitis Biofilms 

**Figure 6:**
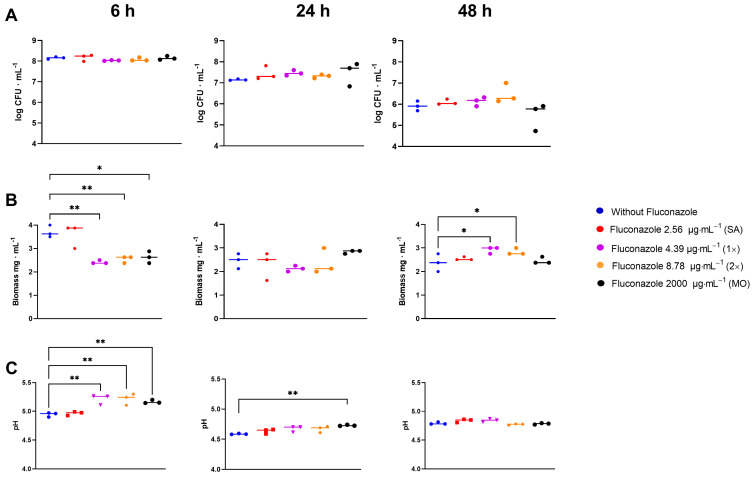
CFU (**A**), dry biomass (**B**) and pH (**C**)of *S. mitis* biofilms grown for 6, 24, and 48 h in the presence of fluconazole at 2.56 μg·mL^−1^ (SA), 4.39 μg·mL^−1^ (1×), 8.78 μg.mL^−1^ (2×), and 2000 μg·mL^−1^ (MO), or without fluconazole exposure (negative control). Statistical difference * *p* < 0.05, ** *p* < 0.01 compared to the control group using One-Way ANOVA followed by Tukey’s multi-comparison post hoc test.

In the *S. mitis* biofilm assay, fluconazole had no significant effect on CFU counts at any time or concentration (*p* > 0.05) (Figure 6A). However, fluconazole significantly altered the biofilm dry biomass by initially reducing it at 6 h, in parallel with a reduced drop in pH, followed by stabilization by 24 h and subsequent increase by 48 h compared to the control samples (Figure 6B). In the presence of fluconazole, the pH dropped slower for the three highest concentrations compared to the negative control at 6 h. At 24 h, the pH for the highest concentration was still higher than the control, and at 48 h no significant differences were observed (Figure 6C).

## 4. Discussion

Antimicrobials can have unwanted side effects related to their impacts on the host cells or non-targeted microorganisms. By using an ex vivo human microbiota model that reproduces the large species diversity found in oral biofilms [25], we demonstrate fluconazole’s effects on the biomass and viability of oral microbial communities in the absence of host factors. Effects on selected streptococcal and non-streptococcal strains were also observed, highlighting the potential of fluconazole to have direct collateral effects on off-target microbes.

The reduction in biomass and increased CFU levels in the oral microbiota model were not accompanied by changes in pH or total DNA load in the microbiota samples. Although the four factors are inter-linked, each methodology requires specific interpretation. Dry biomass measurements report the total weight of dehydrated biofilms, which is influenced by the amount and type of microbes and also by the extracellular matrix fraction. The CFU, on the other hand, provides relevant information on viable cells able to grow under specific growth conditions. Total DNA gives, in turn, an estimate of microbial load, but it does not differentiate between live and dead cells, or between intracellular and extracellular DNA. Overall, the results highlight the complexity of phenotypes influenced by fluconazole exposure. In particular, the increased viability concomitant with decreased biofilm biomass in the fluconazole-treated microbial community samples indicates a potential impact on microbial composition or microbial interactions, possibly affecting community functions towards a reduced production of extracellular matrix components. Our results also showed higher pH values in the supernatant fraction of the wells where the biofilm communities were exposed to fluconazole than in the controls. This occurred despite the increased number of viable cells. This may, again, be associated with changes in composition and/or interactions affecting community functions. In this respect, oral biofilms comprise a large range of bacterial species, some with high acidogenic and aciduric potential, while others can produce copious amounts of alkali. [37] Changes in composition were supported by the results showing changes in the proportion of *V. atypica*, *V. dispar*, *Lactobacillus*, and *F. nucleatum*. In vivo, a variety of additional factors implicated in microbial community responses are expected to influence microbiota composition, including conditions associated with the use of fluconazole, such as, for instance, denture-related stomatitis by *Candida* spp., as well as numerous host and environmental factors. Thus, the model used in our study is not intended to extrapolate the results to in vivo conditions, but to highlight that bacterial communities can be affected by fluconazole by mechanisms other than its antifungal activities. This is in line with our quantitative PCR results that indicated that fungal species, if present, represented a minimal fraction, as they were below detection level using a sensitive assay capable of detecting picomole concentrations of fungal DNA. To our knowledge, this is the first study to investigate the potential antibacterial impact of fluconazole on complex human-derived microbiomes and on oral species. For the single-species experiments, the choice of *S. mitis* was due to its relevance as one of the most prevalent and abundant species in oral microbiomes, and of *S. mutans* was due to its symbiotic association with *Candida* spp. [38]. Non-streptococcal species were further evaluated to better understand the specificity of the effects in single-species models. Future studies examining the potential impact of fluconazole on other species are warranted.

The most studied class of antimicrobials are antibiotics. Antibiotic stress triggered by sub-inhibitory concentrations has been shown to enhance biofilm formation by different bacteria, including streptococci and other major bacterial human colonizers [39,40]. Recent studies indicate that antifungal agents in low concentrations can have a parallel effect on fungi, by influencing fungal biofilm formation [41]. In our model, the concentration of fungal DNA extracted from the microbiota and analyzed by RT-PCR was below the minimal value for reliable detection in (<20 fg) both for controls and fluconazole-treated samples. The finding that bacteria constituted the large majority of the population led us to hypothesize that the fluconazole effect could be related to a direct off-target effect on bacteria. We then investigated the potential antibacterial effect of fluconazole using *Streptococcus mitis*, the most prevalent and abundant streptococcal colonizer of the oral cavity, and *Streptococcus mutans*, an important pathogen in dental caries.

By combining standard culture density measurements and a luciferase reporter system that enables non-disruptive analyses of the metabolic status and viability of streptococci in real time [27,31], we found that fluconazole had no or only a minimal effect on *S. mutans* planktonic growth, whereas for *S. mitis*, there was a concentration-dependent inhibition in growth and in viability/metabolic activity. More than any other concentration, 2000 μg·mL^−1^ reduced and retarded the growth of *S. mitis*. The effect on metabolic activity was also supported by time measurements showing a delay in pH drop in the supernatants of *S. mitis* biofilms exposed to fluconazole. Additionally, the inhibitory effects on *E. coli* and *G. adiacens* planktonic growth and the lack of significant effects on *L. crispatus* and *L. salivarius* growth indicate that the effect of fluconazole can vary for different bacteria and can affect both Gram-positives and Gram-negatives. The molecular mechanisms underlying this matter remain unknown and future investigations should address whether such effects extend to other bacterial species and strains.

Like the oral microbiota biofilm model, exposure to fluconazole resulted in a reduction in biofilm mass by *S. mitis*, but only at the initial phase of biofilm formation (6 h). At 48 h, most of the samples grown in the presence of fluconazole had a higher biomass than the control. Although a direct comparison between the human microbiota model and the single-species model used in our study is not possible given the inherent methodological differences, the results indicate that fluconazole has the potential to impact both complex and single-species microbial communities.

Azole antifungals work by altering the fungal cell membrane via the potent inhibition of cytochrome P450 lanosterol demethylases in the membranes of fungi. In bacteria, P450 was initially found in the Actinomycetes group, including *Mycobacterium tuberculosis*, other mycobacteria, and selected *Streptomyces* species [18]. The binding of azoles to a P450 enzyme of *Mycobacteria* has been demonstrated for fluconazole and several other azoles [18]. With the advancements in next-generation sequencing in more recent years, it has become apparent that P450 enzymes are more widespread in bacteria than previously realized [42,43]. In a large screen within the *Firmicutes* group, the presence of P450 was found in 24% of the species [43]. However, their functions in bacteria and whether any of these can be targeted by azoles remains largely unexplored. An exception is the selected species in the *Actinomycetes* group described above, for which strong inhibition has been demonstrated in different studies [18]. In streptococci, one of the most prominent colonizers of the oral cavity, P450 is not found in any of the species. Since *S. mitis* apparently lacks P450 enzymes, the fluconazole effects observed in *S. mitis* would involve alternative targets. 

Fluconazole’s resistance breakpoint concentrations for *Candida* spp. were recently investigated for their antibacterial effect against selected Gram-positive and Gram-negative species [44]. While quaternary ammonium derivatives of fluconazole had a potent inhibitory effect [44], none of the bacteria tested were inhibited by fluconazole alone. However, the experiments were based on standard methods for MIC determination that examine inhibition only when the bacteria have reached the stationary phase of growth. Another study showed that in plate agar assays, fluconazole inhibited *S. lividans*, although to a lesser extent than imidazoles [18]. Our results indicate that although the effects on *S. mitis* were not observed at the stationary phase, they were present at earlier growth phases. Accordingly, measurements of the density and viability of *S. mitis* revealed a delay in reaching the stationary phase in the presence of fluconazole. Such inhibitory effects would have been missed by standard single-time methods to determine MIC values.

Fluconazole has the ability to permeate various bodily fluids, including saliva [15], and owing to its extended elimination half-life [45], it increases the duration of potential interactions with unintended microorganisms not originally targeted. The effects of long-term fluconazole exposure on this community are still unclear and should be addressed in future studies. Nevertheless, our results suggest that fluconazole may cause an imbalance in the microbiota, and this effect may be more pronounced at the high fluconazole concentrations close to those used in mouthrinses. In addition, the results suggest that fluconazole at clinically relevant concentrations can selectively impact the biofilm and planktonic growth of selected species, and therefore act as a microbial stressor. Numerous environmental stressors, including antibiotics, have been associated with increased biofilm formation and responses that induce resistant phenotypes in bacteria. The lack of studies examining fluconazole’s effects during bacterial growth indicates that the effect of fluconazole as a stressor deserves future attention. Further knowledge about such effects will contribute to a better understanding of the dysbiosis and resistant phenotypes induced by antimicrobials, with potential implications for the development of superior azole-based inhibitors. 

## Data Availability

Data available on request. The data presented in this study are available on request from the corresponding author.
